# Role and Perspective of Molecular Simulation-Based Investigation of RNA–Ligand Interaction: From Small Molecules and Peptides to Photoswitchable RNA Binding

**DOI:** 10.3390/molecules26113384

**Published:** 2021-06-03

**Authors:** Daria V. Berdnikova, Paolo Carloni, Sybille Krauß, Giulia Rossetti

**Affiliations:** 1 Department of Chemistry–Biology, Organic Chemistry II, University of Siegen, Adolf-Reichwein-Str. 2, 57076 Siegen, Germany; berdnikova@chemie-bio.uni-siegen.de; 2 Institute for Advanced Simulation (IAS-5), Forschungszentrum Jülich, 52425 Jülich, Germany; p.carloni@fz-juelich.de; 3 Institute for Neuroscience and Medicine (INM-9), Forschungszentrum Jülich, 52425 Jülich, Germany; 4 Institute of Neuroscience and Medicine (INM-11), Forschungszentrum Jülich, 52425 Jülich, Germany; 5 Department of Physics, Faculty of Mathematics, Computer Science and Natural Sciences, RWTH Aachen University, 52056 Aachen, Germany; 6 Department of Neurology, Faculty of Medicine, RWTH Aachen University, Pauwelsstraße 30, 52074 Aachen, Germany; 7 Institute of Biology (Human Biology/Neurobiology), University of Siegen, Adolf-Reichwein-Str. 2, 57076 Siegen, Germany; 8 Juelich Supercomputing Center (JSC), Forschungszentrum Jülich, 52425 Jülich, Germany

**Keywords:** RNA–ligand interaction, molecular dynamics, light-controllable association

## Abstract

Aberrant RNA–protein complexes are formed in a variety of diseases. Identifying the ligands that interfere with their formation is a valuable therapeutic strategy. Molecular simulation, validated against experimental data, has recently emerged as a powerful tool to predict both the pose and energetics of such ligands. Thus, the use of molecular simulation may provide insight into aberrant molecular interactions in diseases and, from a drug design perspective, may allow for the employment of less wet lab resources than traditional in vitro compound screening approaches. With regard to basic research questions, molecular simulation can support the understanding of the exact molecular interaction and binding mode. Here, we focus on examples targeting RNA–protein complexes in neurodegenerative diseases and viral infections. These examples illustrate that the strategy is rather general and could be applied to different pharmacologically relevant approaches. We close this study by outlining one of these approaches, namely the light-controllable association of small molecules with RNA, as an emerging approach in RNA-targeting therapy.

## 1. Introduction

Initially thought to just act as the intermediate between DNA and protein synthesis, RNA is now known to perform essential and diverse functions within cells. It forms part of the ribosome [1], the spliceosome [2], and other complex assemblies. It exhibits catalytic activity [3] and is also involved in molecular chaperoning [4]. RNA functions as a hub, regulating cellular networks mostly by interacting with proteins [5,6]. Disruptions or alterations of the latter often lead to diseases [7]. Thus, interfering with aberrant RNA–protein interactions in diseases provides an exciting opportunity for creating highly innovative therapeutics that may target either proteins or RNAs. This review focuses on the latter.

Antisense oligonucleotides and synthetic RNAs that redirect the cellular RNA interference machinery or activate CRISPR-based systems have provided a proof of principle for drugs targeting RNA–protein complexes [8]. However, these approaches involve large, often highly charged molecules and present challenges of delivery with regard to crossing the blood–brain barrier, allergic reactions, and poor absorption [9]. Using small molecules to alter RNA function may overcome some of these issues [8,10,11].

Several experimental approaches have provided fundamental insight into RNA–ligand interactions. These include UV–Vis spectroscopy, fluorescence-based methods, isothermal titration calorimetry, and surface plasmon resonance, along with NMR, X-ray and cryo-EM, and chemical probing (for a review, see [12]). However, structure-based drug designs of such ligands are far more challenging to target than other biomolecules such as proteins [8,10,11]. Indeed, RNAs are flexible molecules that can display a large variety of complex secondary and tertiary structures [13]. In particular, RNAs’ structural elements control the specific binding of proteins by creating spatial patterns and alternative conformations for the interactions to occur. Therefore, RNA structures range from fully paired regions to non-canonically paired regions such as hairpins, internal loops, bulges, multibranch loops, and pseudoknots. For these reasons, while analyses of RNA-binding regions of proteins have identified several RNA recognition domains in proteins that can be targeted, protein recognition sites in RNA are less well-understood and difficult to target with traditional rational drug design approaches [14]. 

Molecular Dynamics (MD) simulations significantly help to investigate RNA–ligand interactions [15,16,17], complementing experimental work [18]. MD can shed light on RNA’s structural adaptation/flexibility and molecular recognition [19,20,21] under realistic conditions of an ionic solution [22]. The latter is known to play important roles in RNA stability and intermolecular electrostatic interactions [23]. MD-based enhanced sampling simulations can provide the full free energy landscape associated with ligand binding to RNA as well as can predict the affinity constants [16,17,24]. In a nutshell, MD simulates the atomic motion of (bio)molecules by numerically solving Newton’s equation using an appropriate potential energy function usually referred to as a force field (FF) [25,26]. 

Thus far, MD simulations of RNA in complexes with small molecules have focused mostly on the so-called “hairpin”, a highly pharmaceutically relevant RNA structural element spanning from viruses such as HIV-1 RNA (the so-called transactivation responsive RNA, TAR [27]) to human RNA containing trinucleotide repeat expansions. This review summarizes some of the salient results of these studies. The approach presented is rather general and could be applied to a vast array of ligands. In the conclusion of our review, as an example, we describe ligands involved in light-controllable RNA-targeting therapy.

## 2. Targeting RNA Trinucleotide Repeat Expansions: HTT RNA CAG

Improper RNA folding leads to the dysregulation of cellular processes [13] either by loss of function or gain of function. The former is usually caused by a mutation in sites that are crucial for proper folding and recognition of RNA by regulatory proteins. The latter is instead triggered by the formation of aberrantly folded RNA structural motifs, such as hairpins containing periodically repeating internal loops, in locations where they are not normally present [28]. The most common causes of such pathologies are genetic mutations that lead to inclusions of pathogenic RNA fragments into gene transcripts, such as those observed in nucleotide repeat expansion disorders. RNA repeat expansion is linked to more than 40 human diseases [29] including Huntington’s disease (HD) (RNA CAG expansion), amyotrophic lateral sclerosis (ALS) (RNA G4C2 expansion), and myotonic dystrophy type 1 (DM1) (RNA CUG expansion) [28,30]. These structures can sequester RNA-binding proteins with substantial pathological consequences [31]. 

**Figure 1:**
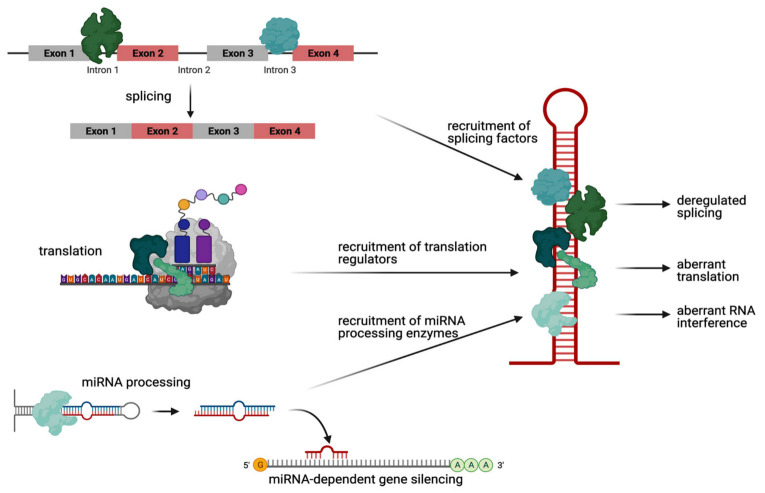
Schematic showing pathogenic mechanisms driven by mutant HTT RNA hairpin structures. More than 40 proteins bind to HTT RNA in a CAG repeat length-dependent manner. Most of them play a role for RNA splicing (upper panel). This recruitment of splicing factors leads to deregulated splicing of the mutant HTT transcript itself as well as to mis-splicing of other mRNA transcripts (upper panel). Some other proteins are instead translation regulators. These, when aberrantly bound to mutant CAG repeat RNA, recruit the translation initiation machinery and thus induce protein synthesis of neurotoxic polyglutamine proteins (middle panel). Finally, few proteins are RNA-processing enzymes of the RNAi machinery. These, when bound to mutant HTT RNA, cleave small CAG repeat RNAs (sCAGs) that finally cause silencing of CTG-containing RNAs (lower panel). Created with BioRender.com (accessed on 23 February 2021).

We focus here on the most known of these diseases, HD. Here, the CAG repeat expansion mutation is located on the huntingtin (HTT) gene. This leads to an aberrant hairpin structure of HTT mRNA with Watson–Crick pairing at the G/C position and intermediate mismatches at the AA position. In addition to these double-stranded structures, these hairpins also form three-dimensional helical structures [32]. The hairpin’s length and stability increase with CAG repeat [33]. Since normal HTT mRNAs do not feature hairpin structures, a relevant role for the RNA structure in inducing a toxic effect is very likely [34]. The expanded CAG repeat hairpin structure is able to bind to and interact with several proteins in a length-dependent manner, thereby contributing to HD pathogenicity (Figure 1) [35,36].

### 2.1. RNA-Mediated Toxicity: Aberrant RNA Hairpin–Protein Interactions in HD

More than 40 proteins bind to HTT RNA in a CAG repeat length-dependent manner, the majority of which are proteins that play a role in RNA splicing [37]. Trapping of the splicing factors leads to abnormal splicing on two levels, either affecting the physiological target mRNAs of the splicing factors or affecting the mutant HTT transcript itself. For example, the splicing factors PRPF8 and MBNL1, when sequestered by mutant HTT RNA, lose their normal function on their physiological target RNAs, leading to mis-splicing of these transcripts [37,38]. In contrast, recruitment of the splicing factor SRSF6 leads to deregulated splicing of the CAG repeat transcript itself, resulting in a mutant HTT from exon 1 to exon 2 [39,40,41], the level of which correlates with the onset of behavioral and neuropathological phenotypes in mouse models [42]. Interestingly, recent studies suggest that this splicing does not depend on SRSF6 alone but that other splicing factors are involved as well [43].

A second well-established group of proteins that are recruited to mutant HTT RNA are translation regulators. Specifically, a protein complex containing the E3 ubiquitin ligase MID1, when aberrantly bound to mutant CAG repeat RNA, recruits the translation initiation machinery and thus induces protein synthesis of neurotoxic polyglutamine protein [44,45]. In detail, MID1 mediates the binding of its complex partners 40S ribosomal S6 kinase (S6K) and protein phosphatase 2A (PP2A) to mutant HTT RNA [44]. MID1 acts as a negative regulator of PP2A [46] and, at the same time, a positive regulator of the PP2A opposing kinase mammalian target of rapamycin (mTOR) [47]. Both MID1 target proteins, PP2A and mTOR, play important roles in translational regulation by controlling the phosphorylation-dependent activity of S6K. Activated S6K induces its target protein S6, a ribosomal subunit. Consequently, the ribosome is assembled and translation is initiated. Thus, MID1 regulates the translation of its target mRNAs including mutant HTT in mTOR- and PP2A-dependent manners via S6K. This suggests that MID1 is a key regulator in the pathogenesis of HD [44]. Therefore, inhibiting the MID1 complex could be an encouraging mechanism to suppress the increased translation of expanded CAG repeat mRNA in HD.

A final group of RNA-binding proteins sticking to mutant CAG repeat RNA are RNA-processing enzymes of the RNAi machinery. Specifically, the ribonuclease Dicer, when bound to mutant HTT RNA, cleaves small CAG repeat RNAs (sCAGs) of <21 nucleotides [48,49]. These sCAGs are then loaded onto the RNA-induced silencing complex (RISC), finally causing a silencing of CTG-containing RNAs [50,51,52].

**Figure 2:**
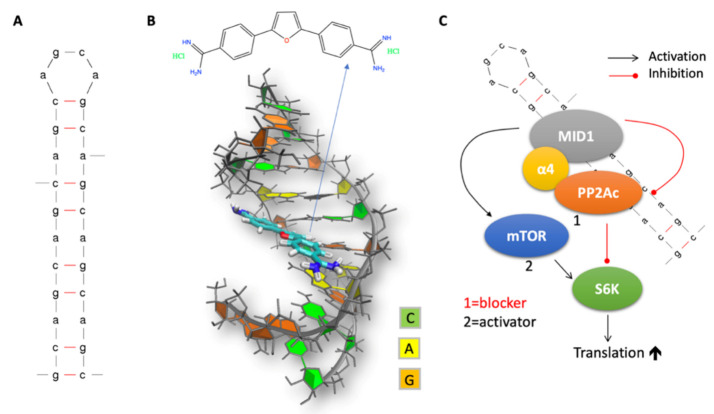
(**A**) CAG RNA hairpin 2D structure. (**B**) CAG RNA hairpin 3D structure in complex with furamidine, as obtained from Reference [17]. (**C**) Scheme of MID1-dependent aberrant translation. MID1 recruits translation regulator proteins including PP2A and S6K to CAG RNA hairpin structures, thereby promoting their translation. MID1-dependent translation initiation is mediated by the opposing activities of PP2A and mTOR on S6K.

In summary, the CAG repeat expansion mutation in HD leads to an aberrant hairpin structure (Figure 2A) that is the basis of the non-physiological RNA–protein complexes involved in diverse cellular mechanisms including aberrant translation of neurotoxic polyglutamine protein.

### 2.2. MD Simulations of HTT RNA CAG with Small Molecules

We show here how the combinations of advanced molecular simulations approaches and experiments led to the identification of RNA CAG binders able to counteract the pathogenic effects of aberrant RNA CAG hairpin–protein interactions in living cells.

Several advanced approaches were conducted experimentally to characterize ligand binding to RNA [53,54]. Computational approaches, such as docking and molecular dynamics, combined with experiments have also been widely used in drug design [55]. However, while the binding pocket of proteins usually lie in an internal region sufficiently separated from solvents, in RNA the binding pockets are usually large and flat, located along the surface, and relatively exposed to solvents [56]. Therefore, properties of RNA such as conformational flexibility, high negative charge, and solvation might challenge both docking algorithms and molecular dynamics approaches [56,57]. Many of the currently available docking programs, initially designed for protein/protein or protein/ligand docking, have been adapted or reparametrized to enable RNA–ligand docking (i.e., Dock6 [58], ICM [59], or AutoDock [60]). A few programs have also been optimized specifically for docking ligands to RNA (i.e., MORDOR [61] rDock (formerly: RiboDock) [62]). Additionally, standalone scoring functions have been designed specifically for RNA–ligand complexes with the intention of being used for rescoring models generated by molecular docking (see for instance DrugScore^RNA^ [63]. In this portfolio of methods facilitating the prediction of 3D RNA–ligand structures, molecular dynamic simulations (MD) also started to have a prominent role. 

MD simulations require an energy model known as a force field (FF). Building a reliable FF for RNA faces challenges. Indeed, RNA FFs require accurate balancing of hydration effects against stacking, base pairing, and many other types of H bonds [21,64]. 

The first all-atom RNA FFs in both the AMBER lineage (ff94) and CHARMM lineage (C22) were introduced in two seminal papers more than a quarter of a century ago [65]. These FFs turned out to be less accurate overall than those used in protein simulations, and several artifacts were observed in RNA simulations [66]. 

Over the last few years, AMBER (used also for all the applications shown here) was substantially improved [21] (i) by refining the backbone and glycosidic torsion parameters [65,67,68,69,70,71,72]; (ii) by finely tuning the van der Waals parameters of nucleobase atoms [73]; (iii) by refining electrostatic, torsional, and van der Waals parameters of atoms other than those in (ii) [74,75,76]; (iv) by using the advanced water FFs, such as the TIP4P–D water model [77]; Although this may not be sufficient to significantly improve the performance of the FFs [21], the choice of water model affects accuracy in the prediction of experimental data such as NMR data [78]; and (v) by developing accurate nonbonded parameters for hydrogen bonding [79,80], which include induced polarization [64]. All of these improvements have succeeded in boosting RNA AMBER FF toward a predictive power approaching that of MD proteins [64,81].

Recent improvements of CHARMM include refining backbone dihedrals, sugar puckering, and glycosidic linkage (i.e., C27 [82,83]) as well as deoxyribose sugar puckering (C36 [84]). 

Polarizable FFs, such as AMOEBA [85] and Drude [86], explicitly account for electronic polarization. They can tremendously boost the predictive power of MD simulations of RNA. However, the lower simulation speed of polarizable-FF-based MD, relative to AMBER or CHARMM MD, might currently limit the domain of their applications. In addition, more investigations are needed to further assess their predictive power.

Let us now discuss specifically MD studies on RNA/small molecules complexes. Several excellent contributions have recently appeared (see, for instance, [87] or [88]). This review focuses on applications involving some of the authors. Using the AMBER force field, Bochicchio et al. predicted the binding pose and affinity of two RNA CAG binders, 4-((diaminomethylene)amino)phenyl4-((diaminomethylene)amino)benzoate (hereafter D6) [89] and 6-(4,5-dihydro-1H-imidazol-2-yl)-2-(4-(4,5-dihydro-1H-imidazol-2-yl)phenyl)-1H-indol-3-amine, towards a CAG repeat oligo r(-GG(CAG)2CC-)2 [16]. Simulations suggested a non-intercalating binding mode for D6, with salt bridges between the guanidine tails of D6 and the phosphate backbone, and parallel (β ring) and T- shaped (α ring) π-stacking interactions with two adenines of the CAG repeats. Based on this information, Matthes et al. identified a set of CAG repeat binder candidates by in silico methods. Among these, furamidine (Figure 2B) was able to decrease the protein level of HTT in an HD cell line model, demonstrating for the first time the activity of an RNA binder against mutant HTT protein in living cells. The observed reduction of HTT protein synthesis (Figure 2C) was likely caused by the reduced binding of HTT mRNA to translation [17]. The calculated affinity was consistent with the experimental data measured in the study [17].

## 3. Targeting HIV-1 TAR RNA Hairpin

We saw that mutations in CAG repeat expansion diseases create aberrant hairpin structures that lead to the formation of non-physiological RNA–protein complexes. These induce diverse cellular processing, including an abnormal translation of the mutant RNA [44,45]. Similar mechanisms of the aberrant RNA–protein interactions that occur in genetic diseases upon the mutation of specific transcripts can also take place in other biological contexts such as aberrant viral-RNA–host protein interactions in RNA-based viruses. These include Orthomyxoviruses, Hepatitis C Virus (HCV), Ebola disease, SARS, influenza, polio measles, and retroviruses including adult Human T-cell lymphotropic virus type 1 (HTLV-1) as well as Corona virus and human immunodeficiency virus (HIV) [90]. 

In such viral infections, hairpin motifs may be involved in the translation of viral RNAs by the host’s ribosomes. Ligands that could block these aberrant RNA–protein interactions would be beneficial in the context of antiviral therapy.

### MD Simulations of HIV TAR with Ligands

Molecular dynamics simulations and free energy calculations, as described above, can obviously be applied to viral RNA as well. 

**Figure 3:**
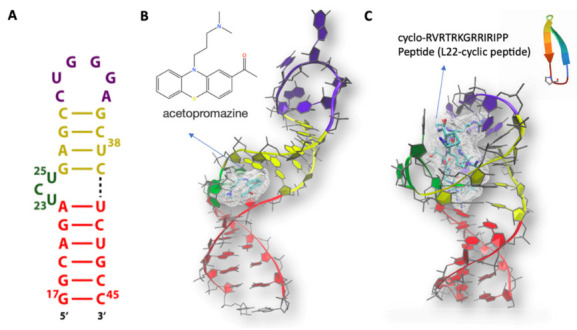
(**A**) HIV TAR secondary structure. The apical loop, bulge, and the upper and lower stems are colored in magenta, green, yellow, and red, respectively. (**B**) NMR structure (PDBID 1LVJ [95]) of HIV TAR in complex with 1-[10-(3-DIMETHYLAMINO-PROPYL)-10H-PHENOTHIAZIN-2-YL]-ETHANONE (Acetylpromazine). (**C**) NMR structure of HIV TAR (PDBID 2KDQ [97] in complex with L-22 CYCLIC PEPTIDE. The same color code as in A is used in B and C for HIV TAR.

Here, we focus on HIV-1’s genome. This genome contains several structural elements, including hairpins, that have been successfully targeted with small molecules to inhibit viral replication [56]. Among these, one of the best characterized RNA-based regulatory machineries is the 59 nucleotide(NT)-long HIV-1 transactivation responsive RNA (TAR) (Figure 3A). Several molecular dynamics simulations have characterized TAR conformational ensemble and dynamics [19,82,84,91,92,93]. One of them, based on the AMBER force field [19], was performed by some of us. Our results appeared to agree well with the NMR data, including residual dipolar couplings and S2 order parameter S2. Calculations of the same system using a different force field turned out to perform more poorly [19,91], highlighting the importance of choosing an appropriate computational setup.

TAR activates elongation of the transcription of the virus by forming a complex with both the virally encoded Tat protein and human cyclin T1 [27]. Indeed, compounds that interact with TAR and prevent formation of the complex could lead to powerful therapies against viral infection. A particularly good strategy is to use Tat mimics (i.e., peptides mimicking Tat binding interactions). In an early study, ligands that could target TAR and inhibit Tat were discovered by the virtual screening of chemical libraries. RNA flexibility was introduced with Monte Carlo simulations and optimized scoring functions [59,94]. The NMR structure of one of them, acetopromazine (Figure 3B), was remarkably different from those with other ligand and Tat. This corroborated the finding that this particular RNA can adopt rather different conformations upon the binding of different ligands. The molecule turned out to completely inhibit the interaction of TAR and Tat at 100 nM [95]. Later, Al-Hashimi’s group developed an outstanding technology for RNA-targeted virtual screening through intensive generation of an ensemble of TAR RNA conformers and a robust re-docking validation test. Their strategy was effectively applied to the virtual screening of a relatively small-sized chemical library containing 51,000 compounds. They identified netilmicin, a selective HIV-1 TAR RNA binder, that inhibited HIV-1 replication in vivo [96]. 

The studies above, along with many other NMR studies [95,97,98,99,100,101,102,103,104], showed that TAR binds to Tat mimics and other small ligands and peptides mostly through its “bulge” (consisting of single stranded nucleotides) with nucleotides n 23–25 (Figure 3A) separating the bulge from the following two helical regions (“upper” and “lower” stems) and that these ligands mostly bind selecting sparsely populated but pre-existing conformations. These studies also showed that conformational fluctuations sustain and assist ligand binding [19] and that the binding of proteins and small molecules alter dramatically the structure and the distribution of ions around the RNA molecule [105,106]. 

One of the peptides used experimentally, the cyclo-RVRTRKGRRIRIPP peptide (referred to as L22 hereafter), mimics the TAR-binding region of the Tat protein [105]. It binds to the bulge area, leading to an increased structural rigidity of the biomolecule [105]. MD simulations on the TAR/L22 complex provided insight into the ligand binding process as well as information on the changes in hydration and counterion distributions that occur upon complex formation [105]. The ions turned out to be displaced from the two molecules (even if they are at long distances from each other) and the peptide and RNA were able to spontaneously bind to each other within the first few nanoseconds of simulation [105].

By building on this research, a method was developed to predict the pose and affinity of highly charged molecules binding to RNA [107]. This work anticipated the crucial role of the ionic clouds surrounding RNA for ligand binding, emerging from recent solution x-ray scattering at wide angles coupled with MD studies. These showed that RNA duplexes have an uneven distribution of cations around their surface, relative to the similar DNA duplex [108]. This asymmetry leads to local structural changes of the RNA (where a strong correlation between cation binding and major groove widening was observed) and to both spontaneous bending and transient binding site formation [108].

## 4. Conclusions and Outlook

More and more pathogenic mechanisms at the base of human diseases are recognized to be connected with aberrant toxic RNA–protein interactions. The general concept underlying RNA-mediated toxicity is that a disease-linked RNA aberrantly recruits RNA-binding proteins, which results in a toxic gain of function of the RNA in conjunction with the protein. Interestingly, increasing evidence has shown that actually similar pathogenic pathways may occur across a variety of diseases. Thus, similar pathogenic mechanisms could be involved in genetic diseases, in which mutant transcripts aberrantly trap RNA-binding proteins, as well as infectious diseases, where aberrant viral RNA–host-protein interactions take place.

Here, we presented a few computational studies of RNA in complex with ligands interfering with the formation of aberrant RNA–protein interactions. The strategy outlined for our case studies, namely CAG repeat expansion diseases and HIV-1 TAR, can be transferred in a straightforward manner to other RNAs and RNA–protein complexes involved in diseases, with a major benefit of reducing time and efforts in the wet lab.

Molecular dynamics of RNA simulations have progressed dramatically in quality and scope over the past four decades, providing important atomistic insights into RNA structure, dynamics, and biological response to cellular and small-molecules modulators. These successes have been driven by the development, assessment, and progressive refinement of the atomistic force field, which in turn have benefited from growing repositories of experimental and quantum mechanics (QM) target data [109].

Benchmarks for RNA FFs have been published in reviews [21,109]. The accuracy of FFs for small-molecule/RNA complexes has not been systematically assessed. Therefore, their validation against experimental data (such as MR and Thermodynamic data [16,17,105,107]) as prior predictions are recommended.

In conclusion, MD simulations approaches are valuable tools to predict the pose and affinity of ligands targeting RNA along with RNA structural plasticity in response to ligand binding [106,108]. The latter is crucial to identify binding pockets that can be exploited for drug design [110]. We envisage future perspective of MD for the development of innovative RNA-targeting approaches. As an example of the latter, we mention here light-controllable RNA–ligand interactions. Among potential external stimuli for drug activation, light is particularly beneficial as a non-invasive, bio-orthogonal, and readily addressable tool acting with high spatiotemporal resolution. Photocontrol on the RNA–ligand binding is especially attractive because of the possibility for the reversible regulation of drug activity and, therefore, reversible manipulation of RNA–protein interactions. Such an approach would be valuable if only temporary switching the transcript off is needed for treatment as well as for the reduction of putative side effects. To the best of our knowledge, there is only a single example of targeting naturally occurring sequences (TAR and RRE-IIB HIV-1 RNA) with a photoswitchable ligand reported up until now [111,112]. However, the selectivity towards certain RNA structures as well as towards the photocontrol of binding still was not achieved. The application of MD here might significantly facilitate the selective design of a photoswitchable pair “RNA-binder/non-binder” targeting certain therapeutically relevant RNA sequences.
